# Growth and Proliferation of Renal Cell Carcinoma Cells Is Blocked by Low Curcumin Concentrations Combined with Visible Light Irradiation

**DOI:** 10.3390/ijms20061464

**Published:** 2019-03-22

**Authors:** Jochen Rutz, Sebastian Maxeiner, Eva Juengel, August Bernd, Stefan Kippenberger, Nadja Zöller, Felix K.-H. Chun, Roman A. Blaheta

**Affiliations:** 1 Department of Urology, Goethe-University, D-60590 Frankfurt am Main, Germany; Jochen.Rutz@kgu.de (J.R.); SebastianMaxeiner@gmx.de (S.M.); Eva.Juengel@unimedizin-mainz.de (E.J.); Felix.Chun@kgu.de (F.K.-H.C.); 2 Current address: Department of Urology and Pediatric Urology, University Medical Center Mainz, D-55131 Mainz, Germany; 3 Department of Dermatology, Venereology, and Allergology, Goethe-University, D-60590 Frankfurt am Main, Germany; bernd@em.uni-frankfurt.de (A.B.); Kippenberger@em.uni-frankfurt.de (S.K.); nadjazoeller@netscape.net (N.Z.)

**Keywords:** curcumin, renal cell cancer, tumor growth, tumor proliferation, cell cycling

## Abstract

The anti-cancer properties of curcumin in vitro have been documented. However, its clinical use is limited due to rapid metabolization. Since irradiation of curcumin has been found to increase its anti-cancer effect on several tumor types, this investigation was designed to determine whether irradiation with visible light may enhance the anti-tumor effects of low-dosed curcumin on renal cell carcinoma (RCC) cell growth and proliferation. A498, Caki1, and KTCTL-26 cells were incubated with curcumin (0.1–0.4 µg/mL) and irradiated with 1.65 J/cm^2^ visible light for 5 min. Controls were exposed to curcumin or light alone or remained untreated. Curcumin plus light, but not curcumin or light exposure alone altered growth, proliferation, and apoptosis of all three RCC tumor cell lines. Cells were arrested in the G0/G1 phase of the cell cycle. Phosphorylated (p) CDK1 and pCDK2, along with their counter-receptors Cyclin B and A decreased, whereas p27 increased. Akt-mTOR-signaling was suppressed, the pro-apoptotic protein Bcl-2 became elevated, and the anti-apoptotic protein Bax diminished. H3 acetylation was elevated when cells were treated with curcumin plus light, pointing to an epigenetic mechanism. The present findings substantiate the potential of combining low curcumin concentrations and light as a new therapeutic concept to increase the efficacy of curcumin in RCC.

## 1. Introduction

Cancer is the second most common cause of death in Europe. The incidence of renal cell carcinoma (RCC) is, compared to other cancer types, relatively rare, but both incidence and mortality are steadily increasing at a rate of approximately 2% to 3% per decade. Already, approximately 3% of all adult cancer patients suffer from malignant kidney tumors [1,2]. RCC is the most frequent form of kidney neoplasm, comprising about 90–95% of all renal melanomas [3]. Approximately one third of these patients have metastases at diagnosis, and 30–70% of patients with localized disease relapse within 5 years of surgery [4]. Since metastatic RCC (mRCC) is resistant to most treatments such as conventional chemotherapy or radiation, patients with mRCC can expect a 5 year survival rate of less than 10% [5]. The introduction of targeted agents including the tyrosine kinase inhibitors, sunitinib and sorafenib, and the mechanistic target of rapamycin (mTOR) inhibitors, temsirolimus and everolimus, have substantially improved patient outcome, but these drugs are not curative due to inevitable resistance development during therapy. 

These unsatisfactory therapeutic options have opened patients’ minds to complementary and alternative medicine (CAM) approaches to actively contribute to treatment, coupled with the hope of prolonging survival or even curing their disease. Up to 50% of cancer patients worldwide are applying CAM [6]. However, knowledge about the efficacy of a particular CAM is often limited due to the lack of evidence based studies. This holds true for the phytopharmacon curcumin, a yellow-orange pigment extracted from the rhizome of *Curcuma longa*, commonly known as tumeric. Apart from its use as a food additive and spice, anti-inflammatory and anti-oxidative qualities have been attributed to curcumin. It promotes the function of the immune system and acts as an antioxidant to capture free radicals, protecting living cells [7,8,9]. Recently, anti-tumorigenic effects in several in vitro and in vivo studies have shown that diverse biochemical processes and pathways triggering carcinogenesis are affected and modulated by curcumin [10]. Curcumin has been shown to inhibit cell proliferation, cell cycle progression, angiogenesis, and cell invasion as well as to induce apoptosis by altering the expression level of pro- and anti-apoptotic proteins [11]. Thus, curcumin is considered a promising possible future adjuvant in cancer management [12].

Low bioavailability, however, remains a serious problem with curcumin treatment. Due to poor water solubility, low absorption, and fast metabolization and clearance, a transfer to clinical use is hampered [13,14]. Human trials carried out so far have provided disappointing results [15,16,17]. 

Bernd et al. recently discovered that irradiation of tumor cells with visible light dramatically enhances the antitumor properties of curcumin [18]. Application of curcumin to tumor bearing nude mice followed by visible light exposure resulted in reduced tumor volumes, reduced proliferation rates, and the induction of apoptosis [19]. On the molecular level, inhibition of extracellular regulated kinases 1/2 and epidermal growth factor receptors along with DNA fragmentation and increased cleaved caspase-3 positive cells has been observed [20,21]. The present investigation was designed to determine the in vitro efficacy of curcumin combined with visible light exposure on RCC cell growth and proliferation.

## 2. Results

### 2.1. Cytotoxicity and Apoptosis

**Figure 1:**
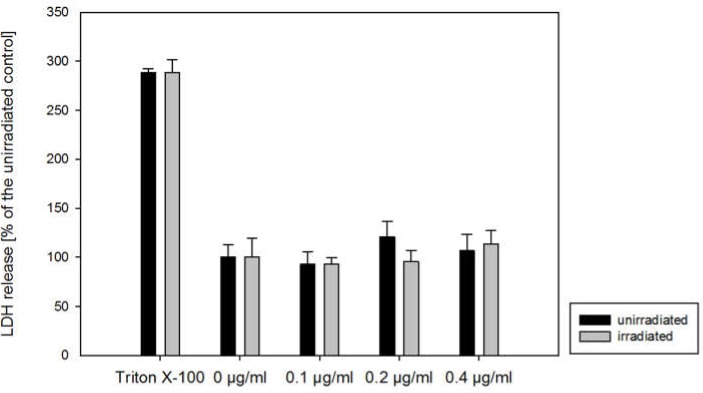
Lactate dehydrogenase (LDH) released from A498 renal cell carcinoma (RCC) cells incubated for 1 h with curcumin (0–0.4 µg/mL) and irradiated with 5500 lx visible light for 5 min (grey) or kept light protected (black). Twenty-four hours later, cell supernatants were prepared. Each column represents the mean ± S.D. of a representative experiment done in triplicate. Cells treated with 1% Triton X-100 served as a positive control.

Curcumin (0.1–0.4 µg/mL) plus light (5500 lx) or curcumin alone did not impair the cell membrane integrity of all cell lines evaluated (Figure 1, representative for A498 cells), as indicated by lactate dehydrogenase (LDH) in the cell free supernatant.

**Figure 2:**
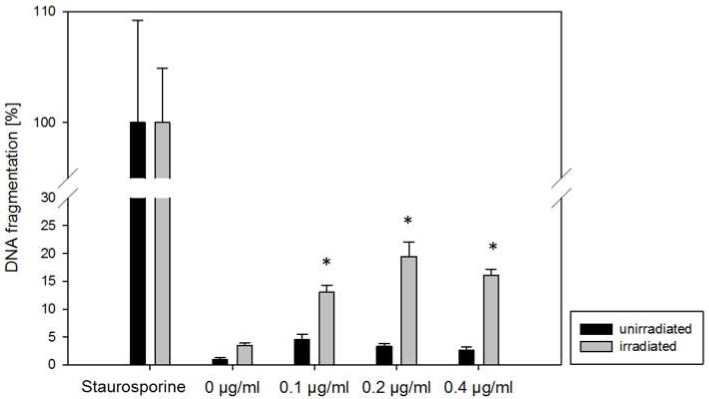
Histone-associated DNA fragments released from A498 RCC incubated for 1 h with curcumin (0–0.4 µg/mL) and irradiated with 5500 lx visible light for 5 min (grey) or kept light protected (black). Twenty-four hours later, cell supernatants were prepared. The positive control was incubated with 1 μM staurosporine (set to 100%). Each column represents the mean ± S.D. of a representative experiment done in triplicate. * indicates significant difference to cells with no curcumin (0 µg/mL).

Curcumin alone, up to 0.4 μg/mL, caused no increase of DNA fragments, as an indicator of apoptosis, in all three cell lines. When visible light was additionally applied, DNA fragments significantly increased in all three cell lines, even at 0.1 μg/mL curcumin (compared to the untreated control; Figure 2: representative for A498 cells, data not shown for Caki1 and KTCTL-26).

### 2.2. Tumor Cell Proliferation

**Figure 3:**
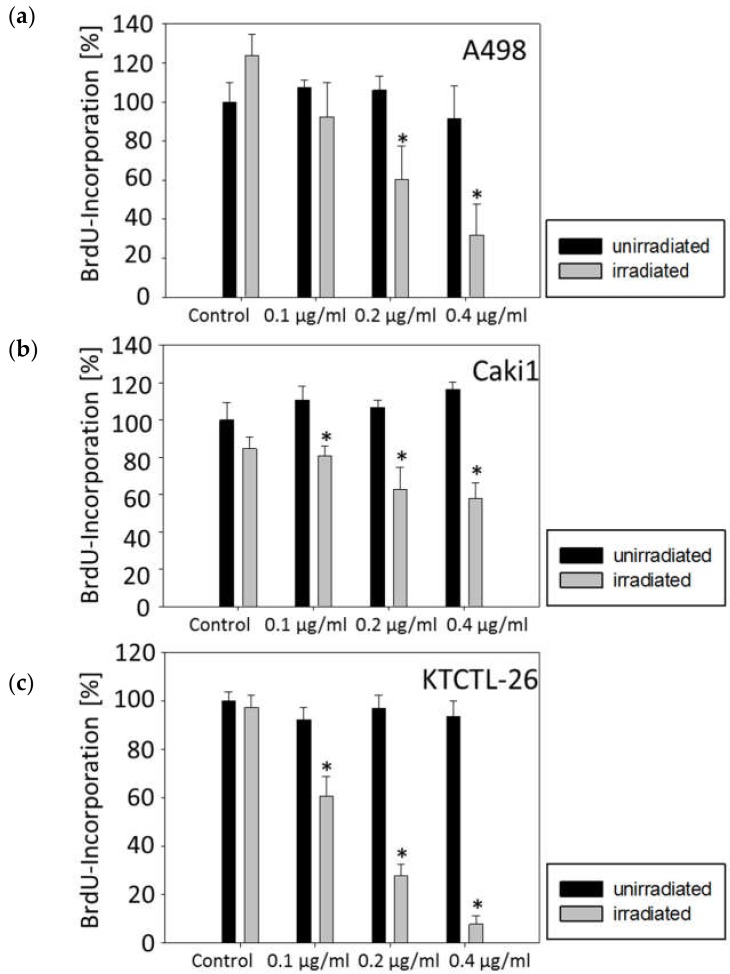
Cell proliferation in A498 (**a**), Caki1 (**b**), and KTCTL-26 (**c**) cells cultured with 0, 0.1, 0.2, and 0.4 µg/mL curcumin without (black bars) or with irradiation (grey bars). Irradiation was done with 5500 lx visible light for 5 min. Tumor cells were then subjected to the BrdU incorporation test following a further 24 h incubation in cell culture medium without curcumin. BrdU-incorporation is expressed as percentage of the untreated cells. Each experiment was done in triplicate and repeated five times. Data from one representative experiment are shown. * Indicates significant difference to controls.

Treating the tumor cells with curcumin and subsequent irradiation evoked a significant dose-dependent decrease of the proliferative activity with maximum effects at 0.4 µg/mL (Figure 3). The most prominent effect was achieved in the KTCTL-26 cell line where the proliferation was decreased by 40% at a concentration of 0.1 µg/mL and up to 90% at a concentration of 0.4 µg/mL. Only the A498 cell line showed no significant proliferation decrease at the lowest concentration of 0.1 µg/mL. Application of visible light alone or curcumin alone led to no significant proliferation differences in any cell line.

### 2.3. Tumor Cell Growth

**Figure 4:**
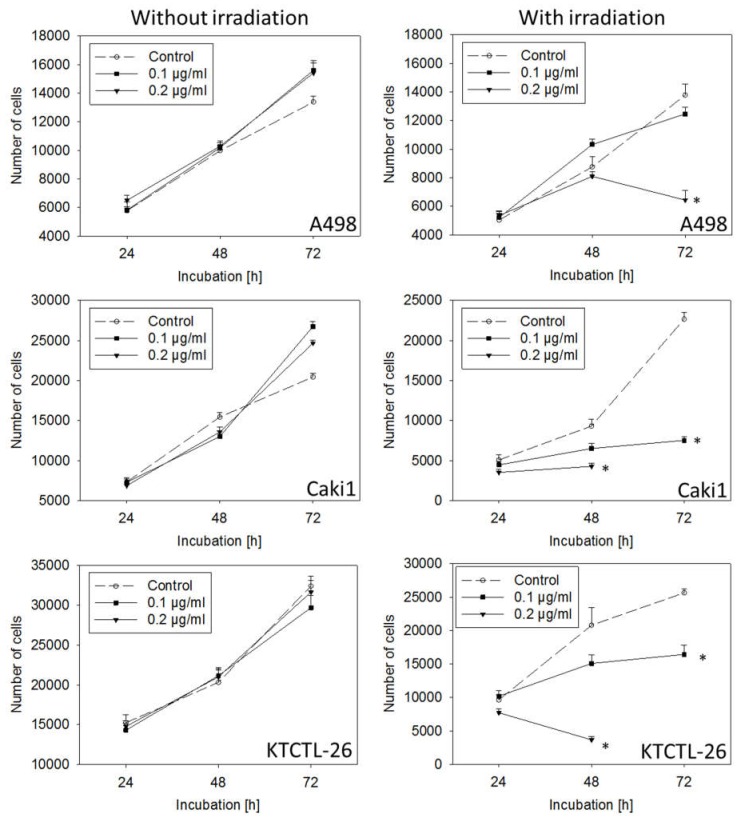
Growth of A498, Caki1, and KTCTL-26 renal cell cancer cells exposed to 0.1 or 0.2 µg/mL curcumin without or with irradiation. Irradiation was done with 5500 lx visible light for 5 min. Following light exposure, curcumin-containing medium was replaced by medium without curcumin. Tumor cell number was then evaluated after 24, 48, and 72 h. Controls remained untreated. Each experiment was done in triplicate and repeated six times. Data from one representative experiment are shown.* Indicates significant difference to controls.

Curcumin applied at low doses of 0.1 or 0.2 µg/mL without irradiation caused no significant alteration in tumor cell number, compared to untreated controls (Figure 4). However, irradiation combined with 0.1 µg/mL (Caki1 and KTCTL-26) or 0.2 µg/mL curcumin (all cell lines) resulted in significantly decreased tumor cell number after 48 or 72 h.

The number of apoptotic A498 cells after 72 h was 21.25 ± 4.33% (0.1 µg/mL curcumin plus light) and 32.06 ± 5.68% (0.2 µg/mL curcumin plus light).

### 2.4. Cell Cycle Progression

**Figure 5:**
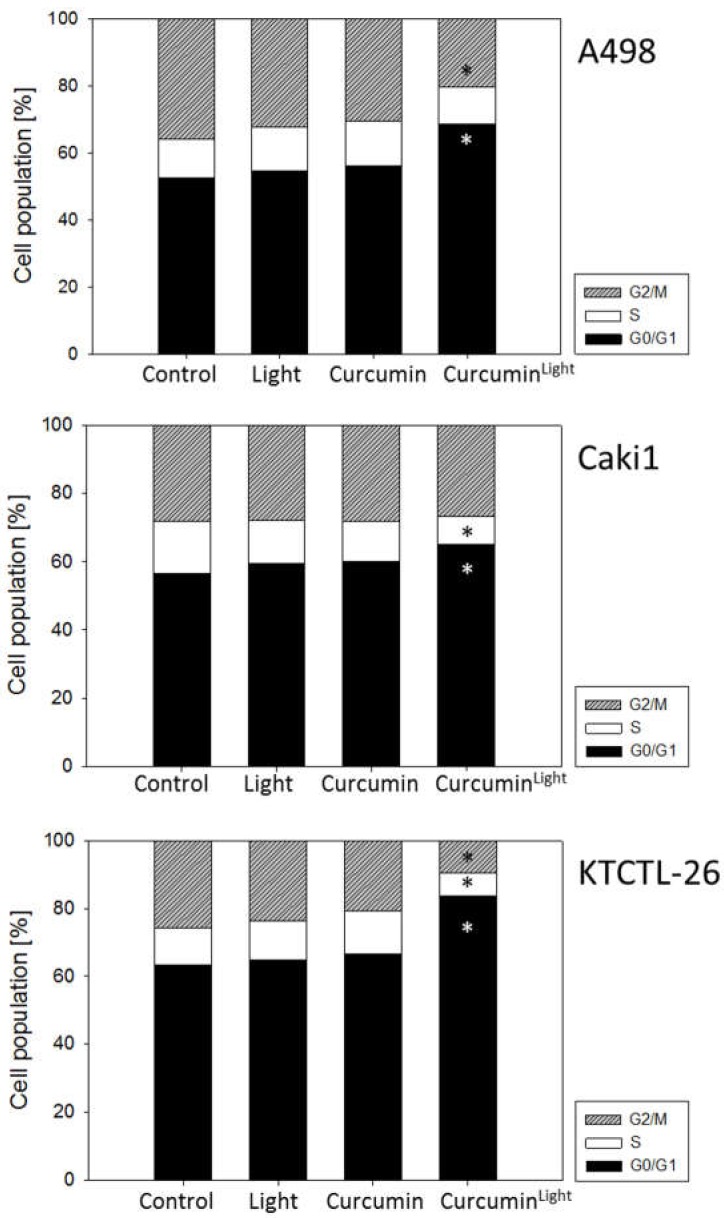
Cell cycle analysis of A498, Caki1, and KTCTL-26 cells treated with 0.2 µg/mL curcumin for 1 h, with or without irradiation (controls remained untreated). Analysis was done 24 h following curcumin exposure. The cell population is expressed as a percentage of the total cells analyzed. One representative experiment of three is shown. Mean SD_interassay_ < 40%, mean SD_intraassay_ < 10%. * Indicates significant difference to control.

Low dosed curcumin combined with visible light induced a cell cycle arrest at the G0/G1-phase (Figure 5). G0/G1-phase arrest was accompanied by a decrease of cells in the S-phase (Caki1 and KTCTL-26) and/or G2/M-phase (A498 and KTCTL-26 cells). Treatment with curcumin or visible light alone did not significantly influence cell cycling. 

**Figure 6:**
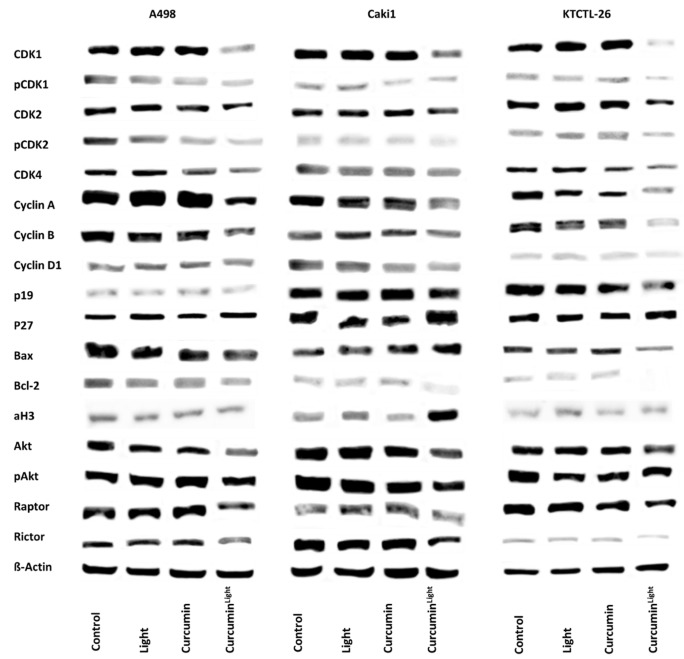
Influence of light, curcumin (0.2 µg/mL), and the combination of both (Curcumin^Light^) on the A498, Caki1, and KTCTL-26 cell cycle protein expression. The protein isolation was carried out 24 h after the respective treatment. β-Actin was used as an internal control. Each experiment was repeated three times. Data from one representative experiment are shown.

The expression of cell cycle regulating proteins is depicted in Figure 6. Exposing the tumor cells to curcumin (0.2 µg/mL) or light did not induce significant changes of the protein expression level, compared to the untreated control cells. However, combining curcumin application with light irradiation, considerably diminished CDK1 and 2 (both total and phosphorylated), together with their respective binding partners Cyclin B and Cyclin A. CDK4 and Cyclin D1 were also reduced, although to a slighter extent, compared to CDK1/2 and Cyclin B/A. The tumor suppressor, p27, was down-regulated in Caki1 and KTCTL-26, whereas p19 was up-regulated in all cell lines analyzed. Bcl-2 decreased in all three cell lines, Bax was diminished in A498 and KTCTL-26, but enhanced in Caki1.

Akt and pAkt were reduced in the presence of curcumin plus light. The mTOR complexes Raptor and Rictor were also diminished by the treatment regimen in all cell lines, compared to controls.

**Figure 7:**
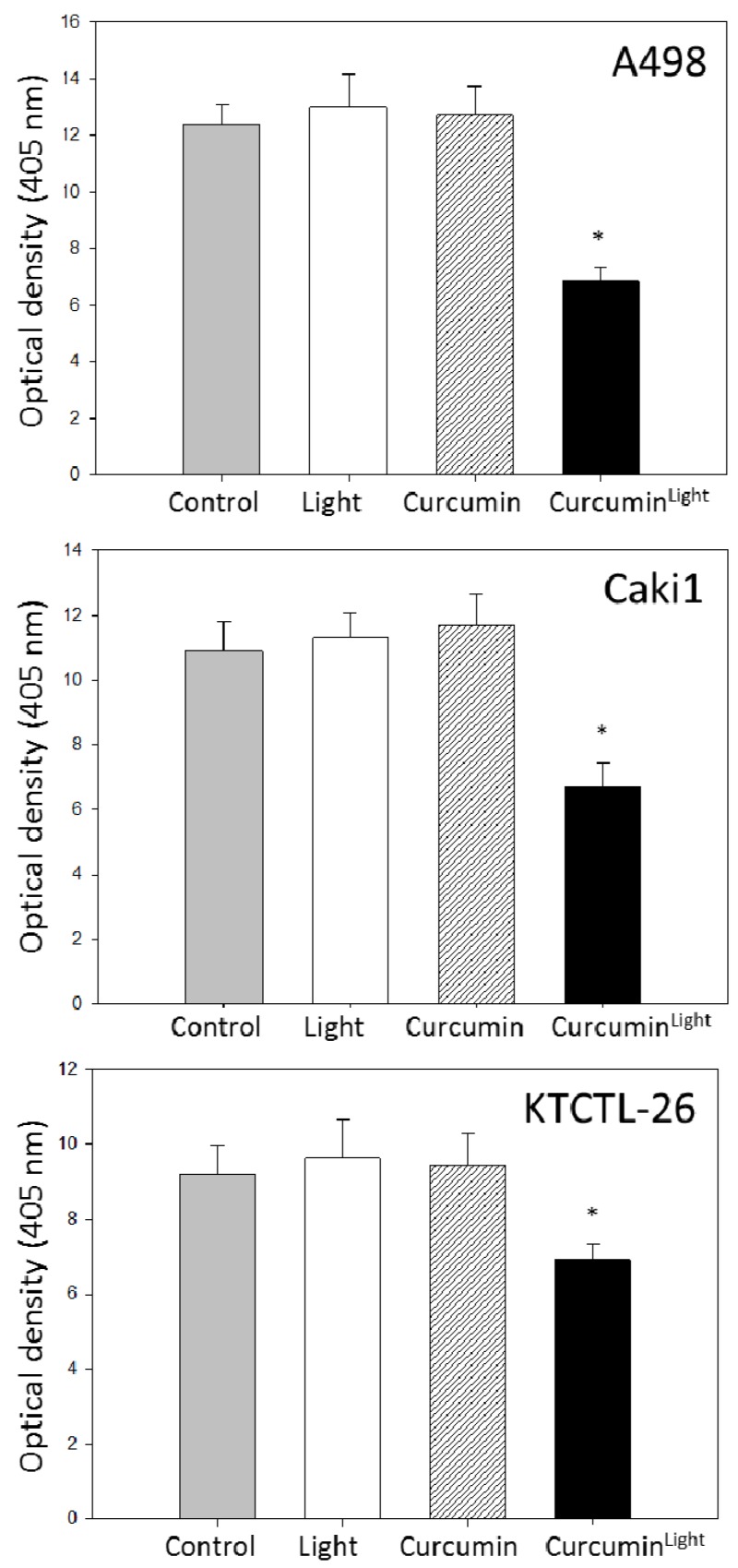
Histone deacetylation activity in A498, Caki1, and KTCTL-26 cell lines. Controls remained untreated (grey) after irradiation (white) or incubation with 0.2 µg/mL curcumin without (hatched) or with irradiation (black). Each experiment was done in triplicate and repeated five times. Data from one representative experiment are shown. * Indicates significant difference to control.

The amount of deacetylated histones was not significantly influenced by light or curcumin alone compared to the untreated control in all three cell lines. However, 0.2 µg/mL curcumin in combination with visible light induced a significant overexpression of aH3 in Caki1 cells, pointing to an epigenetic mechanism. This finding was confirmed by the histone deacetylase (HDAC) expression assay pointing to a reduction of HDAC of about 25% (KTCTL-26), 35% (Caki1), and nearly 50% (A498) (Figure 7).

### 2.5. Knockdown Studies

**Figure 8:**
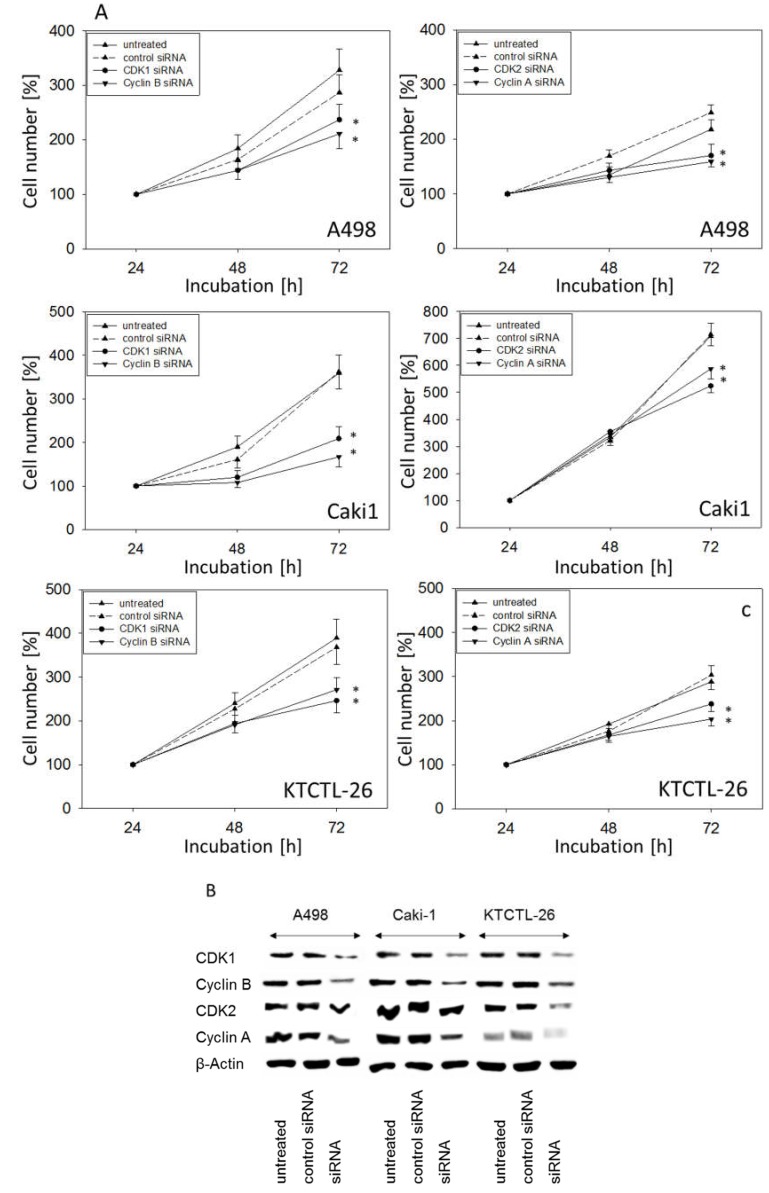
Influence of CDK1, Cyclin B, CDK2, and Cyclin A knock down on tumor cell growth. A498, Caki1, and KTCTL-26 cells were transfected with non-silencing control siRNA, CDK1, Cyclin B, CDK2, or Cyclin A siRNA (**A**). Cell number was set to 100% at 24 h. Knock down was controlled by Western blot (**B**). One representative from six experiments is shown. * indicates significant difference to controls.

Since curcumin strongly modified CDK1 and 2 as well as Cyclin A and B in all tumor cell lines, the physiologic relevance of these proteins was evaluated by siRNA knock-down. Down-regulation of CDK1 and Cyclin B or CDK2 and Cyclin A (Figure 8A) correlated with a significant growth blockade of A498, Caki1, and KTCTL-26 cells. Protein controls are shown in Figure 8B. 

## 3. Discussion

Light exposure greatly increased curcumin’s anti-tumor properties. As low as 0.1 µg/mL (Caki1 and KTCTL-26) or 0.2 µg/mL curcumin (A498) significantly reduced tumor cell growth following irradiation, while 0.1 or 0.2 µg/mL curcumin alone or with light alone did not lead to growth-blocking effects in any of these cell lines. To achieve similar effects on RCC cell growth without irradiation, Caki1 cells required curcumin at a concentration of 2.9 µg/mL (8 µM) (data not shown). This coincides with data from others reporting an IC_50_ of 8 µM curcumin to block the growth of gastric cancer cell lines [22]. Even higher curcumin concentrations have been shown necessary to suppress proliferation of breast cancer (IC_50_: 30–80 µM) [23], neuroblastoma (IC_50_: 30 µM) [24], or bladder cancer cells (IC_50_: 20 µM) [25].

The photodynamic effect is not restricted to RCC cells. A study, using skin keratinocytes, has shown that 0.2 µg/mL curcumin causes a significant proliferation inhibition when combined with visible light. This concentration was more than one magnitude lower than the lowest curcumin (without light) concentration providing pharmacological effects [21]. Curcumin plus light exposure has been shown to inhibit mitochondrial activity in nasopharyngeal carcinoma cell lines, whereas the same concentration without light did not [26]. 

Reduction of cell number was not caused by toxic effects as demonstrated by the LDH assay. However, Bcl-2 decreased in the RCC cell lines treated with 0.2 µg/mL curcumin–light, which may indicate apoptotic events. Indeed, the number of apoptotic cells evaluated after 72 h significantly increased when tumor cells were subjected to curcumin plus light. Dujic et al. have reported a strong increase of apoptotic nuclei in the keratinocyte cell line, HaCaT, 24 h after treatment with light and 1 µg/mL curcumin [21]. On the other hands, DNA fragments did not occur in a melanoma cell model until a curcumin concentration of 0.5 µg/mL was reached [27], and pilot studies on bladder cancer cells have demonstrated signs of early and late apoptosis at 0.4 but not at 0.1 µg/mL curcumin–light (data not shown). Sensitivity to curcumin in terms of apoptosis induction may thus depend on the tumor type.

The analysis of cell cycle progression revealed distinct modulations caused by curcumin. The number of cells in the S-phase (all cell lines) and the G2/M-phase (A498 and KTCTL-26) decreased, whereas the number of G0/G1-phase cells increased. A similar effect has also been observed on breast [23], prostate [28], and lung cancer cells [29]. However, the curcumin effect is not homogeneous. A G2/M-phase arrest has been ascribed to curcumin in a colon cancer [30], a thyroid carcinoma [31], and a colorectal cancer cell model [32].

Based on an investigation by Zhang et al., 10 µM curcumin blocked bladder cancer cells at the S-phase, whereas 15 µM led to a G2/M-phase arrest [25]. The mesothelioma cell line H-Meso-1 was blocked at G0/G1 in the presence of 12 µm curcumin, but stopped at G2/M with 25 µM curcumin [33]. Curcumin’s mode of action, therefore, seems dose associated. Deng et al. recently reported dual effects on autophagy in RCC cells, closely depending on the curcumin concentration [34]. It seems reasonable to assume that the inhibition of RCC growth and proliferation by low-dosed curcumin–light observed in the present investigation is caused by a G0/G1 phase arrest.

Along with the G0/G1 block, the expression of CDK1 and CDK2 with their respective counterparts, Cyclin B and Cyclin A, as well as the Akt-mTOR signaling pathway, was significantly diminished in all cell lines in the presence of 0.2 µg/mL curcumin–light (but not in the presence of 0.2 µg/mL curcumin or light alone). Information about the influence of curcumin on the CDK-Cyclin-axis in RCC is sparse. Diminished expression of Cyclin B has been seen in the RCC cell line RCC-949 following curcumin exposure [35]. Treatment of glioma [36], lung [37], or pancreatic carcinoma cells [38] with curcumin has also been shown to cause a significant reduction of Cyclin B, along with CDK1, and Kuo et al. noted an additional decrease in the protein expression of Cyclin A in nasopharyngeal cancer cells [39]. In all these investigations, curcumin was applied at considerably higher concentrations than employed in the present investigation. In the RCC model from Zhang and colleagues, concentrations up to 100 µM were used [35]. The present data show clear evidence that light exposure to curcumin-treated RCC cells strongly enhances the anti-tumor potency of this compound, whereby alteration of the CDK-Cyclin axis may be only one of several relevant mechanisms contributing to cell growth reduction by curcumin–light. The relevance of the respective CDKs and Cyclins is confirmed here, since protein knockdown significantly blocked tumor growth. 

From a clinical viewpoint, loss of Akt and the mTOR-members, Rictor and Raptor, is of interest. The Akt/mTOR-pathway plays a crucial role in the pathogenesis of RCC, and various drugs targeting this signaling cascade have already been established and approved [40]. Unfortunately, neither the mTOR-inhibitors everolimus nor temsirolimus are able to permanently suppress Akt/mTOR. Rather, resistance develops under chronic therapy, leading to an increase in protein activity and relapse associated with tumor aggressiveness. Integrating curcumin into the oncotherapy might, therefore, optimize the current treatment concept. Combined curcumin and temsirolimus treatment has been shown to exert a synergistic effect on apoptosis in human RCC cells in vitro. The authors of that study concluded that pre-treatment or co-treatment of cells with curcumin might not only enhance the response to targeted drugs, but might also overcome drug resistance in human RCC [41].

The influence of curcumin on HDAC is difficult to interpret. There is little doubt that curcumin targets HDAC and that HDAC-suppression along with histone acetylation may contribute to the anti-cancer effects of curcumin [42]. Based on the present work, only Caki1 cells responded to curcumin–light in terms of elevated aH3, pointing to an epigenetic mechanism in this cell line. It is not clear why A498 and KTCTL-26 did not respond in the same manner. Marquardt et al. discovered that curcumin’s influence on HDAC in liver cancer cells may depend on the extent of inhibition of NF-kB and downstream signaling [43]. Whether this may also hold true for bladder cancer cells remains to be seen. 

The exact mechanism underlying the advantageous effect of light is not totally understood. Speculatively, light-dependent energy transfer during curcumin–protein interactions may enhance the influence of curcumin on protein function and cell regulation [18]. It has been postulated that both the photo-catalytic effect of curcumin and photo-activation are essential triggering factors [44]. A conceivable molecular mechanism of the photo-toxicity of curcumin might also be that curcumin photo-generates reduced forms of molecular oxygen [45]. Regardless of the exact mechanism, the present results demonstrate that combining curcumin with light irradiation could considerably enhance curcumin’s anti-tumor potential. 

Ongoing studies must now deal with the technical aspect of curcumin–light application in renal cancer. Introducing an optical fiber into RCC tumors of a mouse model with subsequent laser illumination of the vascular-acting photosensitizer WST11, either at a single wavelength (750 nm) or multispectrally (700 to 800 nm), induced necrosis in the RCC tissue, as evidenced by histological analysis. [46]. Baran et al. has suggested an interstitial optical fiber-based spectroscopy using sensitizers with high absorption at 780 nm or beyond to optimally treat RCC [47]. mTHPC (meso-tetra(hydroxyphenyl)chlorin), a photosensitizer that targets both vasculature and tissue, has been recommended by others, since its localization in RCC vasculature and tissue may produce a strong combined effect [48]. 

Based on pediatric epithelial liver tumor cell lines, evidence has been provided that irradiation with blue light (480 nm) amplifies the cytotoxic effects of low dosed curcumin. The authors concluded that combining low curcumin concentrations with light irradiation may compensate for low bioavailability and rapid degradation of curcumin in vivo [49]. From a technical viewpoint, irradiation of the tumor bed (including possible invisible micrometastases) with light after tumor resection could take place shortly after curcumin administration. Local laparoscopic light irradiation may be an optional treatment option [49]. Still, further investigation is required to explore whether curcumin specifically acts on the tumor cells or whether healthy tissues and cells may also be damaged by this compound.

Curcumin is not the only compound shown to have enhanced effects when combined with an energy source. Hypericin’s anti-tumor effects have been shown to be enhanced when human RCC cells are exposed to radiation or light in vitro, pointing to a clinical relevance of radiotherapy and intraoperative photodynamic therapy [50]. Ongoing studies must now deal with the technical feasibility of potentiating curcumin’s anti-RCC activity with visible light. The next experimental step will, therefore, be to evaluate the effect of photodynamic therapy after curcumin administration in an RCC in vivo model.

## 4. Materials and Methods

### 4.1. Cell Culture

Renal carcinoma Caki1 and KTCTL-26 cell lines were purchased from LGC Promochem (Wesel, Germany). The A498 cells were derived from Cell Lines Service (Heidelberg, Germany). Caki1 and KTCTL-26 cells were chosen since both lines are derived from a clear cell renal cell carcinoma, which is the most common renal carcinoma tumor type. A498 served as the “classical” RCC cell line used as a model of ccRCC as well [51]. Both cell lines are von Hippel-Lindau (VHL) positive, whereas VHL function is disrupted in A498 cells. The tumor cells were grown and subcultured in RPMI 1640 medium supplemented with 10% fetal calf serum (FCS), 1% Glutamax (all Gibco/Invitrogen, Karlsruhe, Germany), 2% Hepes buffer, and 1% penicillin/streptomycin (both Sigma-Aldrich, München, Germany) at 37 °C in a humidified 5% CO_2_ incubator. Subcultures from passages 5–30 were selected for experimental use.

### 4.2. Drug Treatment and Light Exposure

Curcumin was stored at −20 °C and was diluted in cell culture medium to a final concentration of 0.1–1 µg/mL (0.27–2.7 µM). Cells were treated for 1 h with curcumin and subsequently irradiated with visible light for 5 min (5500 lx, 10×40 W lamps, distance 45 cm, emission spectrum: 400–550 nm, cumulative dose 1.65 J/cm^2^; Waldmann UV 801AL, Villingen-Schwenningen, Germany). 

For irradiation, the cell culture medium was replaced by phenol red free PBS (Sigma-Aldrich). After irradiation PBS was replaced by cell culture medium containing no curcumin. Control cell cultures were exposed to visible light without curcumin, received curcumin without light exposure, or were treated with PBS alone. Tumor cells were then subjected to the assays listed below. 

### 4.3. Cytotoxicity 

Membrane integrity was quantified using a cytotoxicity detection kit (Roche Diagnostics, Penzberg, Germany) based on the release of lactate dehydrogenase (LDH) from damaged cells. Briefly, the cells were cultivated in 96-well plates (2 × 10^4^ cells/0.33 cm^2^) and treated with curcumin and light as aforementioned. The next day, cell-free supernatants were incubated with NAD^+^, which is reduced by lactate dehydrogenase to NADH/H^+^. Consecutively, NADH/H^+^ reduces the yellow tetrazolium salt to a red-colored formazan salt. The amount of red color is proportional to the number of lysed cells. For quantification, the absorbance of the reaction product was measured at 490 nm using an ELISA reader.

### 4.4. Apoptosis

DNA fragmentation was chosen as an indicator of apoptosis. Quantification was performed with the Cell Death Detection ELISA (CDD; Roche, Mannheim, Germany) according to the manufacturer’s instructions. In brief, cells were cultured in 96-well plates (2 × 10^4^ cells/0.33 cm^2^) and treated with curcumin and light as mentioned above. After 24 h, the cytosolic fraction was subjected to a sandwich enzyme-linked immunosorbent assay with the primary anti-histone antibody coated to the microtiter plate and the secondary anti-DNA antibody coupled to peroxidase. Optical density was measured at 530 nm by an ELISA reader.

Expression of Annexin V/propidium iodide (PI) was evaluated using the Annexin V-FITC Apoptosis Detection kit (BD Pharmingen, Heidelberg, Germany). Tumor cells were washed twice with PBS-buffer, and then incubated with 5 µL of Annexin V-FITC and 5 µL of PI in the dark for 15 min at room temperature. Cells were analyzed on a FACScalibur (BD Biosciences, Heidelberg, Germany). The percentage of vital, necrotic, and apoptotic cells (early and late) in each quadrant was calculated using Cell-Quest software (BD Biosciences).

### 4.5. Measurement of Tumor Cell Growth and Proliferation

Cell growth was measured using the 3-(4,5-dimethylthiazol-2-yl)-2,5-diphenyltetrazolium bromide (MTT) dye reduction assay (Roche Diagnostics, Penzberg, Germany). Tumor cells (100 µL, 1 × 10^4^ cells/mL) were plated into 96-well tissue culture plates. After 24, 48, and 72 h, MTT (0.5 mg/mL) was added for an additional 4 h. The reaction was stopped by lysing the cells in a buffer containing 10% SDS in 0.01 M HCl. After incubating the plates overnight at 37 °C and 5% CO_2_, the absorbance at 570 nm was measured for each well using a microplate proliferation enzyme-linked immunosorbent assay (ELISA) reader. Each experiment was done in triplicate. After subtracting background absorbance, results were expressed as mean cell number. 

Cell proliferation was measured using a BrdU cell proliferation ELISA kit (Calbiochem/Merck Biosciences, Darmstadt, Germany). Tumor cells, were seeded into 96-well tissue culture plates, incubated with 20 µL BrdU-labelling solution per well for 8 h, and fixed and detected using anti-BrdU mAb according to the manufacturer’s instructions. Absorbance was measured at 450 nm after 24 h.

### 4.6. Cell Cycle Analysis

Cell cycle analysis was carried out with sub confluent tumor cells after 24 h cultivation with or without 0.2 µg/mL curcumin. Tumor cell populations were stained with propidium iodide, using a Cycle TEST PLUS DNA Reagent Kit (BD Biosciences, Heidelberg, Germany) and then subjected to flow cytometry with a FACScan flow cytometer (BD Biosciences). In total, 10,000 events were collected for each sample. Data acquisition was carried out using Cell-Quest software and cell cycle distribution was calculated using the ModFit software (BD Biosciences). The number of gated cells in the G1, G2/M, or S-phase is presented as %. 

### 4.7. Histone Deacetylation

Histone deacetylation (HDAC) activity of renal cancer cells was quantified using the Color De Lys assay (Enzo Life sciences, Lörrach, Germany) according to the manufacturer’s instructions. Cells were cultivated with curcumin and/or light as aforementioned. All substances were plated on a 96-well plate and the reaction was initiated by adding substrate and stopped by Color De Lys developer. Optical density was measured at a wavelength of 405 nm using an ELISA reader.

### 4.8. Western Blot Analysis

To investigate the level of the cell cycle regulating proteins in the three cell lines, tumor cell lysates were applied to a 7–12% polyacrylamide gel (depending on the proteins) and electrophoresed for 90 min at 100 V. The protein was then transferred to nitrocellulose membranes (1 h, 100 V). After blocking with nonfat dry milk for 1 h, the membranes were incubated overnight with monoclonal antibodies directed against the cell cycle proteins: CDK1/Cdc2 (IgG1, clone 1), pCDK1/Cdc2 (IgG1, clone 44/CDK1/Cdc2 (pY15)), CDK2 (IgG2a, clone 55), Cyclin A (IgG1, clone 25), Cyclin B (IgG1, clone 18), Cyclin D1 (IgG1, clone G124-36), p27 (IgG1, clone G173-524), CDK4 (IgG1, clone 97), p19 (IgG1, clone 52/p19 Skp1; all: BD Pharmingen), pCDK2 (Thr160 Cell Signaling). The mechanistic target of rapamycin (mTOR) pathway was investigated using the following monoclonal antibodies: Raptor (24C12 Cell Signaling), Rictor (D16H9; Cell Signaling), PKBα/Akt (IgG1 clone 55), anti phospho Akt (pAkt; IgG1, Ser472/Ser473, clone 104A282; both: BD Pharmingen). aH3 (Lys9), aH4 (Lys8; both Cell Signaling) and Bax (B-9:sc-7480), Bcl-2 (N-19:sc-492; both Santa Cruz). HRP-conjugated goat anti-mouse IgG and HRP-conjugated goat anti-rabbit IgG (both: 1:5.000; Upstate Biotechnology, Lake Placid, NY, USA) served as the secondary antibody. The membranes were briefly incubated with ECL detection reagent (ECL; Amersham/GE Healthcare, München, Germany) to visualize the proteins and then analyzed by the Fusion FX7 system (Peqlab, Erlangen, Germany). β-Actin (1:1.000; clone AC-15; Sigma-Aldrich, Taufenkirchen, Germany) served as the internal control.

### 4.9. Knockdown Studies of Cell Cycle Regulators

To determine whether CDK1, CDK2, Cyclin A, and Cyclin B impacted tumor cell growth in A498, Caki1, and KTCTL-26 cell lines, cells were transfected with the respective small interfering RNA (siRNA). Per batch, 3 × 10^5^ cells/2.3 mL of medium were transfected with small interfering RNA (siRNA) directed against CDK1 (Hs_CDC2_10, gene ID: 983, target sequence: AAGGGGTTCCTAGTACTGCAA), CDK2 (gene ID: 1017, target sequence: AGGTGGTGGCGCTTAAGAAAA), Cyclin A (gene ID: 890, target sequence: GCCAGCTGTCAGGATAATAAA) or Cyclin B (Hs_CCNB1_6, gene ID: 891, target sequence: AATGTAGTCATGGTAAATCAA) (all from Qiagen, Hilden, Germany) or with an siRNA/transfection reagent (HiPerFect Transfection Reagent; Qiagen) at a ratio of 1:6. Non-treated cells and cells treated with 5 nM control siRNA (All stars negative control siRNA; Qiagen) served as controls. Subsequently, tumor cell growth was evaluated and Western blotting was done as indicated above.

### 4.10. Statistics

All experiments were performed three to six times. Statistical significance was calculated with the Wilcoxon–Mann-Whitney *U* test. Values are expressed as means ± S.D. Differences were considered statistically significant at a *p* value less than 0.05.
